# Options for accounting carbon sequestration in German forests

**DOI:** 10.1186/1750-0680-4-5

**Published:** 2009-08-03

**Authors:** Joachim Krug, Michael Koehl, Thomas Riedel, Kristin Bormann, Sebastian Rueter, Peter Elsasser

**Affiliations:** 1 Johann Heinrich von Thünen Institute (vTI), Federal Research Institute for Rural Areas, Forestry and Fisheries, Institute for World Forestry, Leuschnerstr. 91, 21031 Hamburg, Germany

## Abstract

**Background:**

The Accra climate change talks held from 21–27 August 2008 in Accra, Ghana, were part of an ongoing series of meetings leading up to the Copenhagen meeting in December 2009. During the meeting a set of options for accounting carbon sequestration in forestry on a post-2012 framework was presented. The options include gross-net and net-net accounting and approaches for establishing baselines.

**Results:**

This article demonstrates the embedded consequences of Accra Accounting Options for the case study of German national GHG accounting. It presents the most current assessment of sequestration rates by forest management for the period 1990 – 2007, provides an outlook of future emissions and removals (up to the year 2042) as related to three different management scenarios, and shows that implementation of some Accra options may reverse sources to sinks, or sinks to sources.

**Conclusion:**

The results of the study highlight the importance of elaborating an accounting system that would prioritize the climate convention goals, not national preferences.

## Background

The significance of carbon sequestration by the world's forests and forested landscapes in the climate regime is well acknowledged. Stern described in 2006 [1] the net carbon emissions from forests and forested landscapes by deforestation and forest degradation alone as comprising more than 18% of the global greenhouse gas (GHG) emissions. More recent publications indicate even higher values, e.g. the Worldbank stating in 2007: '... emissions from deforestation and degradation... now account for an estimated 18 to 25% of all global greenhouse gas emissions.' [2].

Specific methodological accounting options, however, are still under debate. Moreover, the limited availability of verified data on carbon stocks and carbon stock changes is another constraint for identifying the implications of the debated accounting options. While the calculation and assessment of past carbon fluxes due to forest management facilitates the reporting of emissions and removals of greenhouse gases under the current accounting framework under the Kyoto-Protocol [3], the prediction of future emissions and removals related to sustainable management activities can provide future guidance for political or scientific measures and initiatives on the provision of incentives for reduced emissions from deforestation and degradation (REDD) as well as for the land use, land use change and forestry (LULUCF) sector in general.

The embedded consequences of accounting options are demonstrated here for the German national GHG accounting. This article presents the most current assessment of sequestration rates by forest management for the period 1990 – 2007 and provides an outlook of future emissions and removals (up to the year 2042) related to three different management scenarios. It also underlines the importance of elaborating an accounting system primarily considering consistency with the climate convention goals rather than considering national preferences.

## Results

### Carbon sequestration by forestry in Germany 1990–2042

**Figure 1:**
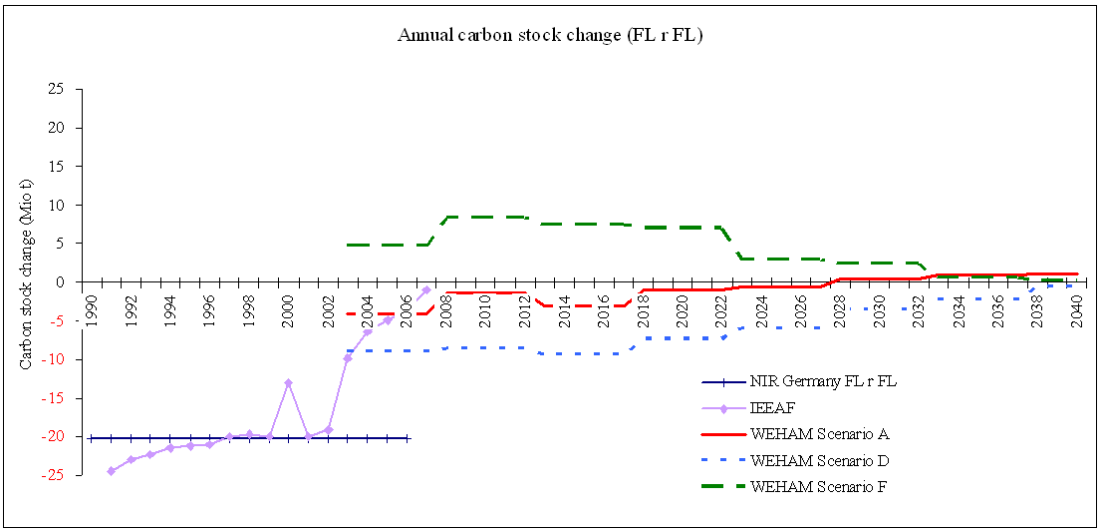
**Annual carbon stock change in Mt C by forest management (excluding afforestation, reforestation and deforestation, ARD)**. Indications from 1990 until 2007 based on IEEAF data and from 2003 until 2040 based on different WEHAM scenarios. The values in 2003 – 2007 reflect a mean between IEEAF and the respective WEHAM scenario. The NIR indicates the officially reported figures to IPCC (extrapolation on constant level). Positive (black) numbers represent emissions to the atmosphere; negative (red) numbers represent removals from the atmosphere (the sink function of forests).

The assessed and predicted sequestration rates by forest management are illustrated in Figure 1 in Mt C/a as developed by the Framework for Integrated Environmental and Economic Accounting for Forests (IEEAF). The elaborated sequestration data are considered as the best available at the current stage and for the presented purpose. The difference between the IEEAF data and the National Inventory Report (NIR, data reported to the United Nations Framework Convention on Climate Change, UNFCCC) can be explained by the lack of more accurate data at the time of reporting: the NIR data assume a constant extrapolation based on two national forest inventories (NFI 1987 and NFI 2002). However, the NIR data are presented here in order to clarify why Germany cannot base a reliable prediction of accounting opportunities on the NIR data unless those are adjusted by more precise information and consider recent changes. For this reason, reference will be made to the IEEAF data compiled as described above.

In Figure 1 the decline in carbon uptake in 2000 is obvious, which reflects emissions related to windthrow caused by the storm 'Lothar' in December 1999. The compensation of those emissions within one year is not only driven by regrowth, but reflects the effects of diverse market-based and management-based adaptation strategies as well. This shows that a single storm event like 'Lothar' may not cause severe compliance risks to Germany thanks to adaptation strategies.

Higher influence on the net emission rates has the continuous reduction of biomass stock from 2002 through 2007 and its consequence on the sequestration rate: the latter was reduced from about 20 Mt C to almost 0 Mt C in five years only. This reduction is mainly caused by market adaptations to growing timber prices but by a reduction of harvestable stands as well. The age-class effect, which results in an unbalanced high ratio of mature stands, is rather supposed to cause a disproportionally high level of emissions by harvesting in the near future. In addition, the combination of two different scenario models implies limited effects.

The different indications for the German national assessment of timber supply (WEHAM scenarios F, A and D in Figure 1) reflect the assumptions as explained above. The current data from IEEAF in 2007 indicate lower sequestration levels as predicted in WEHAM-based scenarios A and D. At the present stage of knowledge it must be assumed that a true development is most likely within in the range of scenario A and F. Scenario D is excluded from further consideration, as even under medium-term accumulation of carbon (biomass) stocks, a forested ecosystem will finally reach a saturation level where biomass gain and biomass loss is in a steady state. Considering a further increasing removal of woody biomass from forests in the near future, the orientation towards scenario F is suggested, which assumes an upcoming reduction of the area of harvestable stands, a requirement by the age-class effect and by related management (stand safety) constraints.

**Figure 2:**
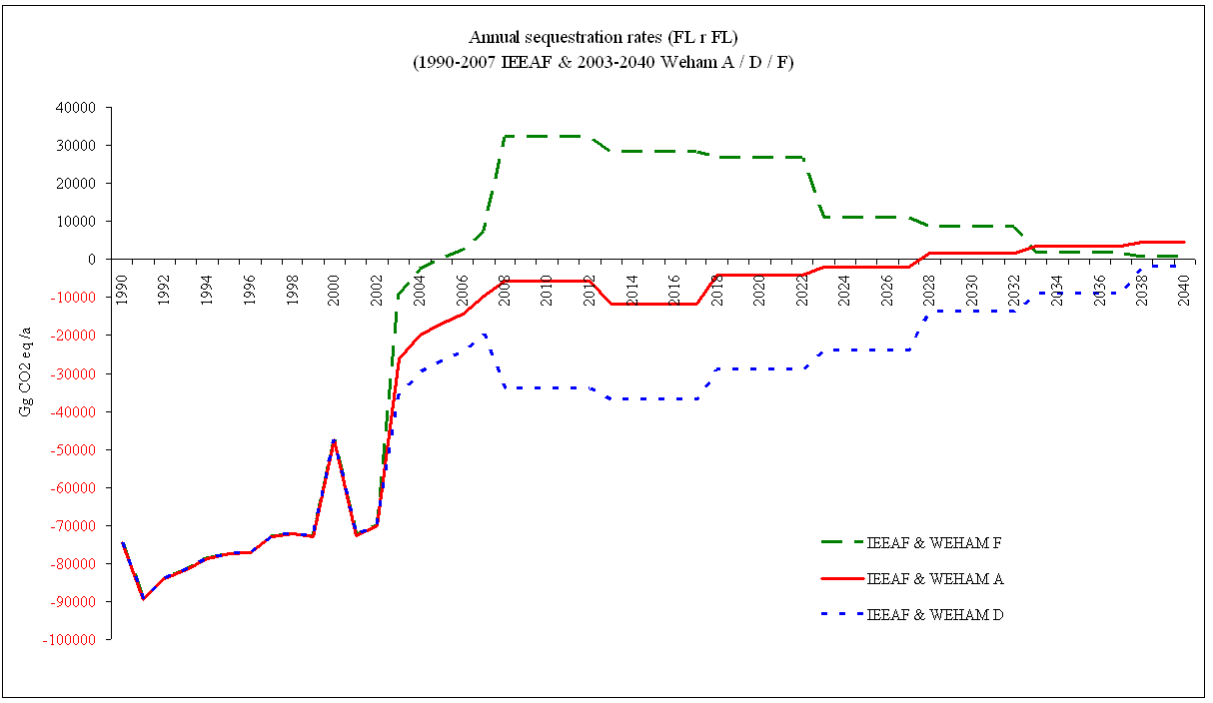
**Annual sequestration rates in Gg CO_2 _eq. by forest management (excluding afforestation, reforestation and deforestation, ARD)**. Indications from 1990 through 2007 based on IEEAF data and from 2003 through 2040 on WEHAM scenarios A, D and F. The values in 2003 – 2007 reflect a mean between IEEAF and the respective WEHAM scenario. Positive (black) numbers represent emissions to the atmosphere; negative (red) numbers represent removals from the atmosphere (the sink function of forests).

Additional market and policy requirements related to harvested wood products for 'clean energy' and for substituting energy-rich building materials underline the expectation of a continuous biomass reduction in the German forests within the near future. In this light, the following will reflect all three WEHAM scenarios, but consider WEHAM-based F as the reference for the prediction of accounting methodologies. Figure 2 presents the annual sequestration rates by forest management (FM) in Gg CO_2_eq./a, where the IEEAF data are combined with the data for the respective WEHAM scenarios.

### Consequences of different accounting options

#### Consequences associated with the choice of a reference level

In past negotiations the specification of a baseline related to a reference year was preferred. Emissions and removals in a fixed reference year may be not in accordance with the long-term levels due to exceptional events such as storm damage or market imbalances. Thus a fixed reference year can stipulate a specific, extraordinary situation and may allocate a-priori losers and winners. Specifying fixed or moving reference periods is an option for levelling out compliance risks.

The consequences of gross-net accounting (GNA) and net-net accounting (NNA) for the German forestry sector can only be explained by a detailed consideration of those respective reference levels for respective base year emissions (BYE) or base period emissions (BPE). However, under any Accra Accounting Option referring to net-net accounting (Accra Option 1B, 2, 3 and 4) of forest management, both approaches, BYE and BPE are applicable.

**Figure 3:**
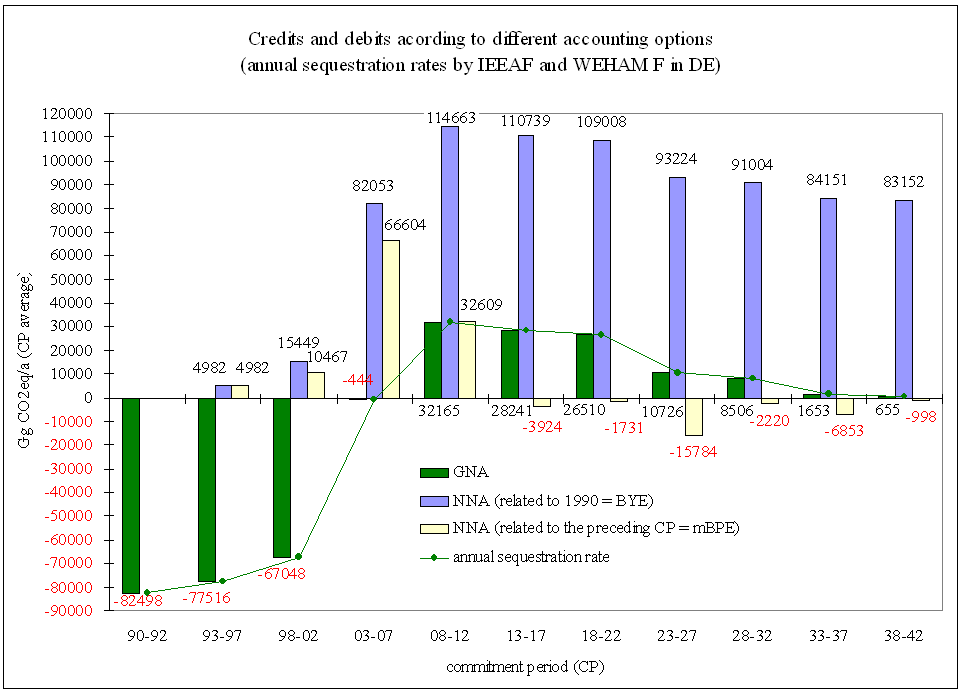
**IEEAF & WEHAM F based gross-net accounting (GNA) and net-net accounting (NNA) related to base year emissions of 1990 and moving base period emissions**. Positive (black) numbers represent emissions to the atmosphere/debits; negative (red) numbers represent removals from the atmosphere (the sink function of forests)/credits.

The emissions figures from IEEAF and WEHAM scenario F presented above are taken as an example to demonstrate the consequences of referring to BYE rather than BPE (Figure 3). The green bars reflect the annual gross sequestration rate as it would be accounted for under GNA. The yellow and blue bars reflect the accountable emissions/removals under NNA related to the base year emission of 1990 and related to the preceding commitment period (CP-1), respectively

In early 1990 Germany and other European countries had to suffer from the heavy damage caused by the storms "Vivian and Wiebke". In order to regulate the timber market and to halt the decline of timber prices, the German Federal Government issued a regulation [4] that limited timber harvest for fir, spruce, oak and beech between 40 to 80% of the regular annual cut for the years 1990 and 1991. As a consequence comparably high sequestration rates were observed in 1990 and 1991. In subsequent years a continuous reduction of sequestration rates was observed, which is assumed as a consequence of the recovery of the timber market. Under GNA, net emissions from FM would need to be reported from the commitment period (CP) 2008–2012 onwards. Under NNA (when 1990 is utilised as the base year) even higher debits for every year would need to be reported. Referring to the base year 1990, which is characterised by an exceptionally low harvest rate, would enforce the reporting of debits for every foreseeable commitment period in every new CP and entail a 'punishment' for not-reaching an unrealistically high sequestration rate; no matter what efforts are taken. As selecting incidentally a specific year as reference for emissions (i.e. BYE) might result in inequitable situations for individual Parties, base period emissions (BPE) were presented as an alternative approach for establishing a reference. Base period emissions are illustrated as moving BPE based on IEEAF and WEHAM F assumptions.

Currently the best choice for a reference selection is discussed by Parties. The desire to recognise latest policy efforts and ambitions in forest management in the current CP's accounting may lead to a preference of selecting a preceding CP instead of a constantly fixed reference year. Parties can take the advantage of levelling compliance risks within the five years of a CP and relate the emissions/removals of the actual CP to values of the preceding CP by providing for e.g. natural disturbances. This guarantees for a flexible accounting system, which makes allowance for non-human induced disturbances and automatically integrates an adaptation component into the accounting system. However, the application of such a moving Base Period Emission (mBPE) referring to the preceding CP bears the risk of so-called "perverse" incentives for forest management regimes that aim at an increase of credits in future CPs. But on a long run the reference to the preceding CP is supposed to yield incentives for a stable level of optimum forest carbon stock; this holds especially true when sustainable forest management regimes are practiced.

Under NNA with mBPE (Figure 3) emissions would have to be reported in the CP 2008–2012, but in preceding CPs emission reductions are to be reported. While the age-class legacy is still visible, in CP 2013–2018 the NNA is related to the considerable emissions in the preceding period (2008–2012). Hence, the improved situation compared to CP 1 would be "rewarded" and qualify for credits. It must be noted however, that such considerations should reflect not only preferable situations for Parties, but must predominantly concern the goals of the convention. Thus, the accounting system must consider the trend in GHG emissions 'as the atmosphere sees it'.

#### Consequences of the integration of a cap and a discount factor

**Figure 4:**
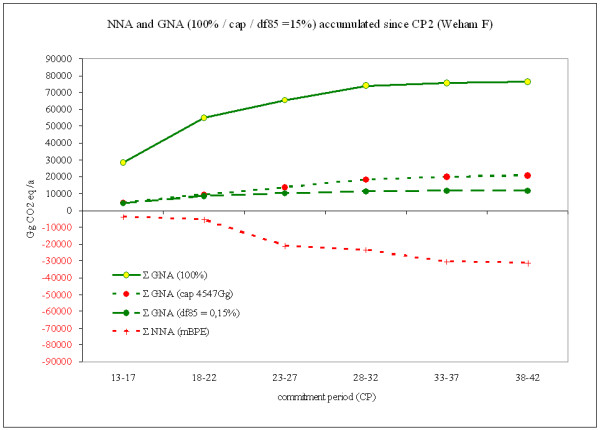
**Accumulated emissions/removals from the second CP onwards relating to WEHAM F based assumptions under GNA applying cap and different discount factors and NNA with mBPE CP-1**. Positive (black) numbers represent emissions to the atmosphere; negative (red) numbers represent removals from the atmosphere (the sink function of forests).

Another important method among the Accra Options is the integration of a cap or a discount factor. The objective pursued by both excludes non-human impacts (windfall profits as well as compliance risks) on LULUCF by limiting the accountable credits and debits from GNA. Another argument for constraining GNA is the avoidance of a 'too easy' compensation of other sectors' emissions by Parties which hold vast forest resources. However, the cap as well as the discount factor introduce limits to accountable credits that do reflect purely political decisions in order to provide for national circumstances (16/CMP.1, Appendix Z [5]) and can be interpreted as volition to attach more or less importance to the land-use and forestry sector. Figure 4 illustrates the accumulated emissions/removals from the second CP onwards relating to IEEAF and WEHAM F assumptions under GNA with cap and discount factor and (for comparative reasons) NNA with mBPE.

The difference between the line for unconstrained GNA and the capped GNA is simply reflecting the magnitude of 'real' (i.e. unconstrained) and constrained accountable emissions and removals. The GNA discounted by a df provides the predominant advantage of allowing incentives beyond the strict limit set by a cap. It reduces all accountable emissions or removals by a fixed proportion, which was arbitrarily set to 85% in Figure 4. By that, the smaller the emissions/removals are, the more the meaning of the LULUCF sector will be reduced. Furthermore, the magnitude of a proportional discount factor directly triggers the incentives for carbon sequestration since the discounted quantity can be directly translated into corresponding revenue losses. In case of high emissions due to disturbances or high harvest rates, accounting is restricted as well. While the Accra Option 1A includes the cap, Option 1B utilises the discount factor. Current discussions suggest a discount factor of 85%, hence allowing an accounting of only 15% of the gross emissions from FM.

For the sake of completeness, Figure 4 includes the estimates for the accumulated NNA mBPE CP-1 approach. Under the given assumptions (IEEAF and WEHAM F), the NNA mBPE approach would generate credits from the second CP onwards. As shown above, the NNA mBPE approach balances compliance risks within certain limits and reduces the accountable emissions/removal by accounting for the additional values of subsequent CPs only. This allows for accounting without artificial rules for capping or discounting. The mBPE approach, however, differs from all other accounting approaches and sectors.

#### Consequences associated with the choice of a net-net accounting approach

**Figure 5:**
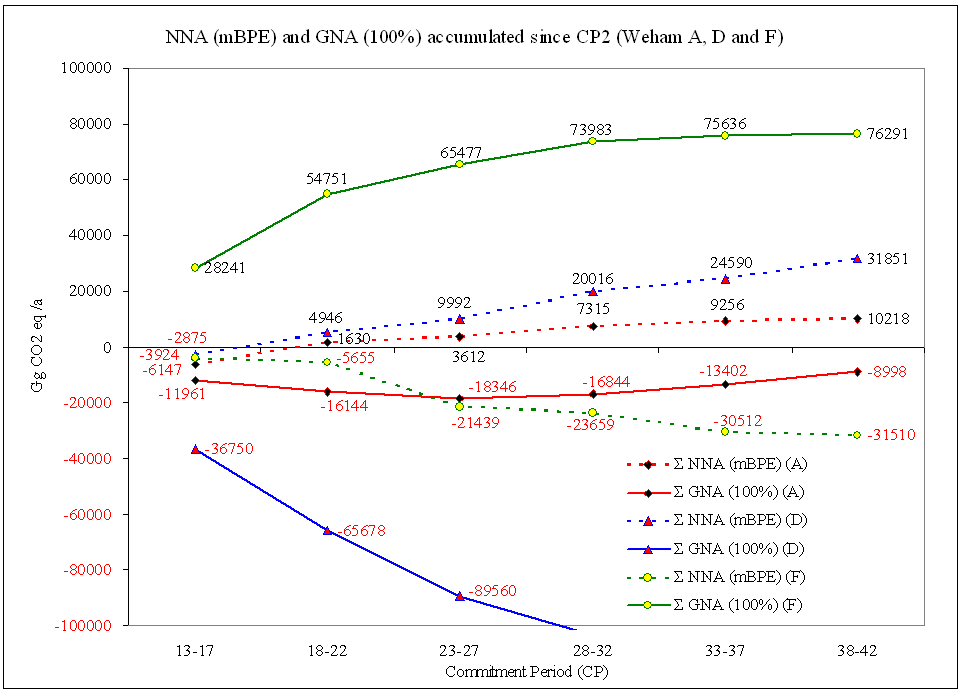
**Accumulated emissions/removals from the second CP onwards relating to WEHAM A, D and F under GNA and NNA with mBPE**. Positive (black) figures represent emissions to the atmosphere; negative (red) figures represent removals from the atmosphere: the forests function as sink. The comparison of three different management options embedded in the WEHAM scenarios and two accounting alternatives shows the wide range of accountable credits.

Under gross-net accounting no reference is made to emissions and removals from past commitment periods, GNA considers only the gross emissions or removals of the current commitment period. Net-net accounting relates the accountable emissions or removals to a defined reference year or reference period, by which a specific level for accountability is introduced and only "additional" efforts to exceed or failures to meet past emission/removal levels are honoured. To facilitate comparisons, Figure 5 illustrates predictions based on the three different WEHAM scenarios, each under unconstrained GNA and NNA mBPE.

While WEHAM scenario D (rotation period extended by 20 years) does provide the highest amounts of credits over time under GNA, it does not provide any credits under NNA. However, the accumulation of such high stock levels as stipulated by WEHAM scenario D is – not only for reasons of stand security – unlikely in the intensively managed forests in Germany.

Under WEHAM scenario A (base scenario), more credits would be accumulated under unconstrained GNA than NNA. In case a cap or discount factor is introduced, those gains by GNA are reduced as demonstrated in Figure 4. WEHAM scenario F (reduced growing stock) is the 'more likely' scenario under consideration of forest management and expected market development. The NNA mBPE under this scenario is the alternative creating most credits for accounting in the long run.

## Discussion

The findings presented above show clearly that the approach utilised for carbon budgeting defines the amount of emissions and reductions from forests that qualify for accounting. It is quite striking to note that approaches do only partly reflect the true situation of carbon sequestration and emissions by forests and may reverse sources to sinks and vice versa.

Further on, the possibility of selecting different activities under Kyoto Protocol (KP) Art. 3.3 and Art. 3.4 allows further manipulation of national emission budgets to be accounted for. While accounting for activities under KP Art. 3.3 (afforestation, reforestation and deforestation) is mandatory, activities under KP Art. 3.4 (forest management, grassland management and cropland management) can be voluntarily applied by Parties. Only the "zero-option", i.e. no more KP Art. 3.4 accounting despite prior election, does not exist unless the overall accounting rules according to decision 16/CMP.1 on LULUCF are changed – which is not to be expected at the current state of negotiations.

"Accra Option 4" aims at dissolving that problem by replacing activity-based accounting with mandatory land based accounting through combining KP Art. 3.3 and Art. 3.4. Such a land-based accounting approach would serve the integrity of the accounting system. As Parties already have to report emissions from all activities, a land-based accounting would not even necessarily imply an additional burden on the reporting system.

## Conclusion

The choice of the different components of an accounting system establishes from the outset the magnitude of emissions and removals that qualify for accounting. Parties might opt for their "optimal" choice by referring to predictions of the future development of carbon stocks in forests and the accountable credits and debits. While those predictions may well represent the future development of forest growing stock based on past evidence there is uncertainty about future political, societal and economic variables and their impact on forest management objectives. An option to absorb uncertainties about future timber utilisation rates is the recognition of harvested wood products (HWPs) and their direct and indirect substitution potential in future accounting schemes. However, in order to avoid "perverse" incentives and penalisation the design of flexible and objective schemes for accounting carbon sequestration in forestry remains a major challenge.

The implications of the different accounting options on the magnitude of accountable net-emissions demonstrated the substantial influence of the methodology on results. The choice of the different components of an accounting system establishes from the outset the magnitude of emissions and removals that qualify for accounting.

This insight again underlines the importance of elaborating an accounting system primarily considering consistency with the climate convention goals rather than considering national preferences.

We hope that the findings presented in this paper may support the elaboration of a broadly accepted accounting system and facilitate political negotiations.

## Methods

### Assessment of the carbon sink capacity of German forests within the European Framework for Integrated Environmental and Economic Accounting for Forests (IEEAF)

Within the scope of Natural Resource Accounting, EUROSTAT, the statistical office of the European Union, has developed a Framework for Integrated Environmental and Economic Accounting for Forests (IEEAF) [6]. The objective of IEEAF is to consistently link forest-related economic activities with the supply and use of wood within the European economies in physical and monetary terms. The IEEAF standard tables also contain carbon balance sheets for woody biomass and for the forest ecosystem and information provided by the Parties.

Carbon balance sheets are derived from the physical timber balance sheets within the IEEAF-Framework [7]. Physical timber balance sheets comprise timber stock accounts, gross increment, total removals and other accounts. For Germany, the physical timber calculations rely partly on model calculations using data from the two National Forest Inventories with reference years of 1987 [8] and 2002 [9], respectively, and on the German national assessment of timber supply (WEHAM). Total harvest data are derived from annual German Economic Accounts for Forestry (FGR), which is part of German National Economic Accounting [10].

In order to compare carbon accounts by IPCC methodologies [11] and IEEAF different approaches for modelling land area of forest land have to be considered. IEEAF land area balance sheets assume annual land area fluctuations, whereas Intergovernmental Panel on Climate Change (IPCC) methodology-based carbon stock data refer to a constant forest land area at a specific base year. For the calculations in this paper, 1993 was used as base year and IEEAF timber accounts were adjusted accordingly. Based on the adjusted timber accounts the carbon balance for German forests is calculated by following the IPCC Good Practice Guidance for LULUFC [11]. A coefficient of 0.5 is utilised to transform timber coarse volumes into timber dry matter. In a next step timber dry matter is converted into carbon applying a conversion coefficient of 0.5, which is the default value according to IPCC definitions [11].

Besides carbon coarse wood dry matter, the carbon dry matter of the so-called other woody biomass is a second component, which comprises roots, small branches and twigs. As direct measurement methods for the volume of other woody biomass are lacking, specific expansion factors are directly applied to carbon coarse wood dry matter in order to estimate the carbon content. The method for calculating carbon balances within the NIR [12] uses expansion factors which require very detailed information about tree species and age classes. These are not available from the IEEAF balance sheets. On the other hand, the method for calculating carbon balances within the NIR is not able to represent annual timber removals.

In order to adjust the NIR expansion factors for their usage of IEEAF balance sheets, at first the ratio of the aggregated carbon dry matter and the volume of standing timber as published in NIR are computed for the two reference years. In a second step, these ratios are disaggregated into different components and the respective conversion factors: volume of coarse wood into dry matter, dry matter into carbon and the expansion factor of other woody biomass to carbon figures. These expansion factors for IEEAF increased slightly between 1993 and 2002, which is a result of an increasing proportion of younger trees above coarse wood, e.g. through forest transformation measures. Expansion factors in IEEAF did not change from 2002 on, since it is not possible to expand the 2002 national forest inventory data to estimates of the future proportion of young trees.

The yearly variation in the amount of carbon sequestrated is caused by the utilisation of annual rather than average harvest data. Those variations however are superimposed by a sharp carbon sequestration decline in the German forests from 2002 onwards. This is attributable to continuously increasing timber harvests and a decrease of the average timber growth increment.

### Projection modelling of forest development and timber harvesting potential (WEHAM)

The WEHAM model ("Projection modelling of forest development and timber harvesting potential") estimates the potential roundwood availability and related potential forest development, especially the growing stock over the next 40 years [13,14]. WEHAM is a single tree model consisting of three sub-models for tree growth, for exploitation/harvest, and for timber assortments, respectively. The growth sub-model is based at data from the two German National Forest Inventories providing data for 1987 and 2002. It is used for extrapolating tree increment on a regional and species' related scale. The exploitation sub-model implements assumptions about parameters such as thinning intensity and frequency, age and the minimum threshold diameter of the final harvest cut. Additionally WEHAM provides an estimate for the growing stock volume of the dominant crop only, but not of the subsidiary stand.

WEHAM allows for specifying assumptions for individual scenarios, e.g. forest management practices. General conditions like climate, selection of tree species, or the forest area with legal restrictions on exploitation cannot be parameterised. The WEHAM-model excludes economic parameters, technical conditions for logging (e.g., slope, forest road density) and tree mortality. This paper presents three potential scenarios (A, D and F) of growing stock development over time, which are based on different assumptions about future forest management (further WEHAM scenarios (B, C etc.) have been developed by [13] and [14] but were disregarded by those authors in early development stages):

#### A) Base scenario

The base scenario (scenario A) refers to assumptions about forest usage which have been developed in accordance with the management objectives of the state forestry administrations. For the next four decades, these are:

1.) a high and nearly constant level of growing stock in private forests,

2.) a growing stock in the state forests comparable to the level of private forests,

3.) a further increase of growing stocks for coniferous tree species (as current stem diameters for spruce and pine in the dominant age classes are below the threshold values for harvesting), and

4.) a decrease of growing stocks for deciduous tree species (as the current diameter stem diameters for beech and oak in the dominant age classes have reached the threshold values for harvesting).

#### D) Scenario with extended rotation period (20 years)

In scenario D, the final exploitation is postponed by 20 years compared to the base scenario and the diameter threshold for harvesting is increased by 10 cm. Scenario D illustrates a development where the forest functions carry more weight in relation to the production function.

#### F) Scenario with reduced growing stock

According to the NFI 2002 the average growing stock amounted to roughly 320 m^3^/ha in 2002. This level exceeds the levels observed in past decades and is well above other European countries. Maintaining such high growing stock levels results in increasing production risks on the one hand, and decreasing roundwood availability on the other. Therefore scenario F describes the potential roundwood availability which will be given when growing stocks are reduced to the level which was observed in the year 1987 (the base year of the first NFI). Accordingly, the rotation periods are reduced so that the growing stock in 2022 will be reduced to the level of 1987.

### Converting WEHAM results to carbon stock

WEHAM projects growing stock aggregated at species group level for entire Germany in periods of 5 years up to 2042. The WEHAM results are taken a) as the basis for modelling the potential development of carbon stock changes in the LULUCF class 'forest land remaining forest land' for the next 40 years in a straightforward way and b) for analysing the consequences of different Accra Options.

In a first step the projected growing stock was converted into tons of biomass, B, using the functional relationship at tree species level [15]



where

B = biomass [tons]

V = volume of coarse wood (> = 7 cm) [m^3^]

r_0 _= gross density [t/m^3^]

β_V _= degree of volume shrinkage

The biomass estimates resulting from NFI and WEHAM, respectively, represent the above ground coarse wood biomass only (cw, diameter > = 7 cm). However, the Good Practice Guidance (GPG) for Land Use, Land-Use Change and Forestry [11] requires the quantification of the total above and below ground tree biomass, which can be estimated by using coarse wood biomass as input parameter (Table 1).

**Table 1:** Conversion and expansion factors for different tree compartments.

**species group**								
spruce	fir	pine	douglas	larch	beech	oak	short rotation broadleaves	Long rotation broadleaves

*Density by volume [t/m^3^] *[12]								
0.3788	0.3629	0.4307	0.4141	0.4873	0.5583	0.5707	0.4618	0.5642

*Mean sw/cw-ratio *[15]								
0.1453	0.2847	0.1541	0.1050	0.0957	0.1929	0.2091	0.1902	0.1929*

*Mean needle/cw-ratio *[15]								
0.0987	0.0987**	0.0871	0.0463	-	-	-	-	-

*Since no separate value for long rotation broadleaves is available, the value for beech is assumed.

**Since no separate value for fir is available, the value for spruce is assumed.

sw = small wood (diameter < 7 cm), cw = coarse wood (diameter ≥ 7 cm)

In order to account for additional compartments of above ground tree biomass, species-specific expansion factors as published in Dieter and Elsasser [16] have been applied for small wood (sw, < 7 cm) and needle biomass. To eliminate a possible double counting, leaves and needles of summer green species have been omitted in this study (since they are part of the organic layer during wintertime). Roots have been included by applying the root biomass expansion functions of the same meta-analysis [16]. In this source, regression coefficients are given for calculating root biomass (bgb) as a function of above ground biomass (agb), where tree species are distinguished by several dummy variables. Strictly speaking, the mentioned expansion functions are valid at stand level rather than at aggregate (national) level; applying them to aggregate biomass data leads to a slight underestimation of total root biomass because it ignores the higher root/above-ground biomass ratio given in forest stands of low above-ground biomass (i.e. younger stands). However, single stand data have not been available. The approach applied here serves the spirit of conservative estimation.

With respect to a further calculation of error budgets, the usual conversion factor from dry matter into carbon of 0.5 given in the GPG [11] is adopted. Although species specific conversion factors would be desirable (e.g. as investigated for American tree species by Lamlom and Savidge [17], we decided against applying such differentiated factors due to two reasons: First, the standard IPCC factor has an associated error estimate, which is very often not available at tree species level; second, reliable conversion factors for the relevant European tree species vary more between the tree compartments than between trees and tree species respectively.

### The 'Accra Accounting Options'

The so-called 'Accra Accounting Options' were assembled in Accra (Ghana, 21.-27. August 2008) by the convened delegates of the AWG KP 6 and reflect the advisable accounting methodologies discussed at that time. Table 2 provides a commented overview.

**Table 2:** The Accra Accounting Options; advantages and disadvantages.

**option**	**Art. 3.3**	**Art. 3.4**	**advantage (+)/disadvantage (-)**
**0 (KP rules)**	mandatory GNA	voluntary, FM: GNA with fixed cap, other 3.4: NNA	+ simple, no complicated accounting rules
			+ uncertainties and disturbances can be left out (voluntary)
			+ almost no incentives for increasing biospheric GHG removals
			- factoring out arbitrarily dealt with by cap
			- unfair treatment of windfalls/liabilities
			- 'voluntary excuse' and cap reduces incentives to do more
			- Complicated rules, different in the LULUCF sector to other sectors

**1**	mandatory GNA	1A: voluntary 1B: mandatory FM: GNA with discount factor, other 3.4: NNA	+ incentives increased by discount factor
			- high opportunity costs for (100-df) stock increase

**2**	mandatory GNA	mandatory NNA	+ stronger incentives for mitigation action
			+ pragmatic factoring out by cancelling out
			+ a base period can diminish the random impact of a base year
			+ HWPs fully accounted
			+ same NNA accounting rules across all sectors
			+ accounting for 'what the atmosphere sees'
			+*(FM for a stable level of optimum forest carbon stock: incentive for SFM up to a certain level)*

**3**	mandatory GNA	FM: NNA with forward looking baseline	+ ex-post adjustment allows factoring out of natural disturbances
			- complicated review of baseline setting and ex-post adjustments
			- unclear methodological process for baseline setting

**4**	land based NNA accounting according to the convention (FL, CL, GL, WL, S, OL)		+ land-based for all managed lands
			+ LULUCF as any other sector
			+ simplification and broader coverage on mandatory basis
			+ reduced uncertainties
			+ remove any perverse incentives arising from partial or inconsistent accounting rules
			- potential of compliance risks and the issue of effects due to natural disturbances, age structure and harvesting cycles

The Accra Options reflect discussions about mandatory or voluntary accounting of KP Art. 3.3 (afforestation, reforestation and deforestation: ARD) and Art. 3.4 (forest management, FM, grassland management, GM, cropland management, CM, and revegetation, RV), gross-net accounting (GNA) and an upper limit by cap or a discount factor, net-net accounting (NNA), the forward-looking baseline (FLB) and the land-based net-net accounting of Art. 3.3 and Art. 3.4 under the convention reporting land categories. For a better understanding of the theoretical consequences, the different methods of the accounting options for forest related activities only are discussed below.

#### Mandatory vs. voluntary accounting

Accounting for activities under KP Art. 3.3 (ARD) is mandatory in general. According to the Accra Options, activities under KP Art. 3.4 (FM, GM, CM and RV) may or may not be mandatory. Under 'Option 0' (KP rules as currently carried out), the 3.4 accounting is voluntary and only Parties which have opted for those have to account the related emissions and removals. There is a controversial discussion among the Parties about the 16/CMP.1 §19 decision made in Marrakesh [5]: a Party which has opted to account for GHG emissions and removals of a unit of land once has to account for the emissions and removals from these lands forever ('once KP land always KP land'-principle).

The main argument around mixing mandatory and voluntary accounting is to leave it open to individual Parties including the emissions and removals from FM, while the main argument for mandatory accounting is to avoid 'cherry picking'. The option to account for FM, CM and GM on a voluntary basis is reduced in the 'Accra Option 1' and dismissed in the remaining alternatives.

Only 'Accra Option 4' provides a different approach: here the activity-based accounting (ARD, FM, CM, GM, RV) is dismissed completely in favour of land-based accounting (forest land, FL, grassland, GL, cropland CL, wetlands, WL, settlements, S, and other land, OL) by combining Art. 3.3 and Art. 3.4.

#### Gross-net vs. net-net accounting

**Figure 6:**
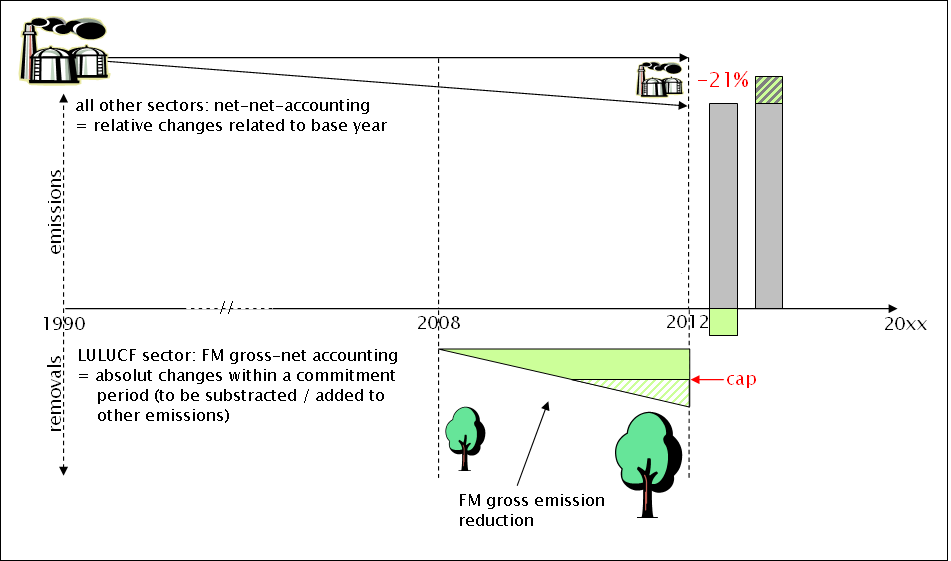
**FM gross-net accounting (GNA) and the cap**. The left bar indicates carbon emissions (grey) and removals (green), right bar indicates total emissions allowable to meet emission targets.

Gross-net accounting is applied to ARD and FM only. Under gross-net accounting (GNA), the gross emissions or removals of the current commitment period are considered as an 'extra' amount that will be added on (or subtracted from) the overall emission of a Party's commitment period (Figure 6); by this potentially helping to meet the national commitment. Under GNA, the emissions and removals from LULUCF are not related to any reference year.

As illustrated in Figure 6, the gross removals from the accounting period 2008–2012 are simply subtracted from the Parties' total emissions, thus allowing compensation for e.g. industrial emissions which must not or cannot be reduced any more. However, to avoid a vast compensation of emissions from other sectors, and to reflect uncertainties and the non-direct human induced change of emissions and removals, the accountable amount of GNA emissions/removals is simply restricted by a national cap (Accra Option '0'). This cap is based on political decisions and reflects only a pragmatic solution (calculated by 85% of discount and lower than 3% of base year emissions of a Party). The current cap for Germany is fixed at 4547 Gg CO_2_eq./a (1.24 Mt C/a).

Under Accra Option 1A and 1B, the cap is replaced by a discount factor. The discount factor (df) is supposed to allow stronger incentives beyond the values of capping. For further discussion of the principle it is assumed in the following that 15% of the total GNA emissions/removals from FM will be accountable, which translates into a df of 85%.

Net-net accounting (NNA), in contrast, is always relating to a reference level or base line. Such a base line may be base year emissions (BYE), like the emissions in the year 1990. An alternative is the relation towards base period emissions (BPE) like e.g. the emissions/removals accounted for in a previous accounting period. Thus, the net emissions of a commitment period are set into relation to those of the base year or base period. The difference between both net emission values are to be accounted for.

**Figure 7:**
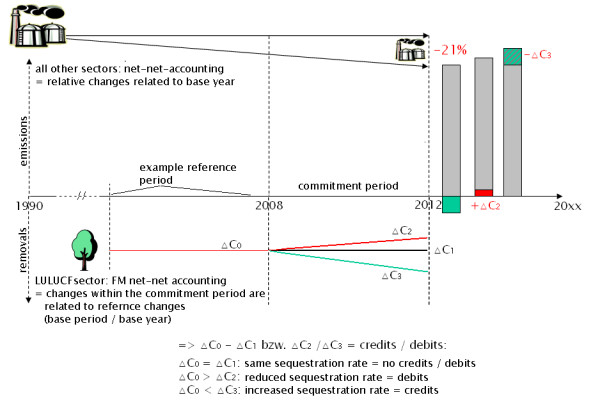
**FM net-net accounting (NNA) under constant sequestration rates**. The left bar indicates carbon emissions (grey) and removals (green), middle bar indicates emissions including emissions from forests (red), right bar indicates total emissions allowable to meet emission targets.

Figure 7 illustrates the removals by carbon sequestration for a hypothetical reference period at 'C_0_'. Within the commitment period 2008–2012, three different scenarios are illustrated: assuming a constantly maintained sequestration rate at the same level ('ΔC_1_') would result in the same net emission rate: ΔC_0 _= ΔC_1_. Hence, no credits or debits will be accounted for. The reason for this is that only additional efforts are to be rewarded for factoring out natural sequestration impacts. On the other hand, this implication can lead to the rather disagreeable situation of not-punishing constant deforestation or degradation rates – or, in contrast, it can lead to not-rewarding constant carbon sequestration as long as the removal or sequestration rates remain at the same level as in the baseline.

In the case of a reduced sequestration rate (ΔC_2 _< ΔC_0_), debits are created in spite of the fact that the LULUCF sector is still functioning as a sink – but a sink that is of lower magnitude. Only in the case of a further increased sequestration rate as compared to the base line (ΔC_3 _> ΔC_0_), debits are accounted for. In the same line, the emission targets of the other sectors are reduced by ΔC_3 _or increased by ΔC_2_.

The NNA would have to be applied similarly in the case of emission rates, here as well including the hypothetically disaccording situation of 'not punishing' a constantly maintained emission rate (e.g. by constantly ongoing deforestation).

However, the NNA is understood to enable the provision of better incentives than GNA, simply because it rewards additional efforts only. Thus, it allows for a better potential of factoring out non-human-induced activities. Another argument in favour of the NNA approach is its applicability to REDD and all the other sectors; this fosters the internal consistency of the LULUCF sector with the entire system.

Further consideration is required for specific circumstances associated with the LULUCF-sector. One important circumstance is the age-class legacy, recognising unbalanced age-class distributions within various Parties. Unbalanced age-class distributions may result a-priori in huge emissions or removals in certain periods without direct human-induced impact (the 'human-induced impact' is supposed to be related to post-1990 activities only, thus not considering consequences of pre-1990 activities). In that understanding, the NNA can limit the effect of an age-class legacy to a certain extent.

Further on, the LULUCF sector is certainly more affected by extreme natural disturbances than other sectors. Such age-class legacy and potential extreme natural disturbances (e.g. forest fires, pests or storms) are considered to threaten the accounting of FM with serious compliance risks. One option for reducing such compliance risks is to utilise a base period rather a base year as reference for the current CP under NNA. Selecting the preceding commitment period as reference level (moving base period emissions, mBPE) will level the impact of age-class legacies and extreme natural disturbances to a more tolerable magnitude. The underlying implications will be demonstrated for Germany in the following figures.

#### The forward looking baseline (FLB)

The FLB approach (Accra Option 3), as proposed e.g. by Canada, specifies a future baseline that is to incorporate estimated amounts of non-human induced emissions/removals as well as expected compliance risks, such as emissions from forest fires, or extreme natural disturbances like insect calamities or draught. The ex-post adjustment of the FLB allows the correction of the emissions and removals which have to be reported according to significant changes towards the previous estimate. The FLB allows for both, an ex-ante inclusion of compliance risks and factoring out and an ex-post adjustment afterwards.

In theory, this could be a fairly smooth way of handling those undesirable emissions and non-direct human induced removals. However, the practical implementation is more difficult, there is still no consensus met concerning the eligibility of scientific methodological approaches for producing sound figures for the FLB approach. IPCC concluded 2003 that it is not possible to factor out non-direct human induced emissions and removals in a sound scientific way. In view of the pronounced variability between individual WEHAM scenarios presented above, the serious (if not dominant) impact of the timber market on the carbon stocks and sequestration rates becomes obvious. While it might be feasible to provide model-based predictions of carbon stocks and sequestration rates, an ex-ante prediction including the driving factors market and policy is arguable. For this reason there is a wide consensus that the FLB is not to be considered any further unless the underlying methodological problems are solved.

## Abbreviations

agb: above ground biomass; ARD: Aforestation; Reforestation, and Deforestation; Bgb: below ground biomass; BPE: base period emissions; BYE: base year emissions CP: commitment period; FL, GL, CL, WL, S, OL: forest land, grassland, cropland, wetlands, settlements, and other land; FLB: forward looking baseline; FM, GM, CM and RV: forest management, grassland management, cropland management and revegetation; Gg CO_2_eq.: Gigagram CO_2_-equivalent (1 Gg = 1000 t); GHG: Greenhouse gas; GNA: gross-net accounting; GPG: Good Practice Guidance; HWP: harvested wood product; IEEAF: Framework for Integrated Environmental and Economic Accounting for Forests; IPCC: Intergovernmental Panel on Climate Change KP: Kyoto Protocol; LULUCF: Land Use, Land Use Change and Forestry; mBPE: moving base period emissions; NFI: National Forest Inventory; NIR: National Inventory Report; NNA: net-net accounting; REDD: Reducing Emissions from; Deforestation and Degradation; UNFCCC: United Nations Framework Convention on Climate Change; WEHAM: Waldentwicklungs- und Holzaufkommensmodellierung (German national assessment of timber supply).

## Competing interests

The authors declare that they have no competing interests.

## Authors' contributions

JK assembled the contribution. WEHAM scenarios were compiled by TR, the IEAAF calculations were worked out by KB and PE. SR and MK significantly contributed to the content. All authors have read and approved the final version of the manuscript.

## References

[B1] Stern N, Peters S, Bakhshi V, Bowen A, Cameron C, Catovsky S, Crane D, Cruickshank S, Dietz S, Edmonson N, Garbett S-L, Hamid L, Hoffman G, Ingram D, Jones B, Patmore N, Radcliffe H, Sathiyarajah R, Stock M, Taylor C, Vernon T, Wanjie H, Zenghelis D (2006). Stern Review: The Economics of Climate Change.

[B2] Worldbank (2007). Two New World Bank Carbon Facilities Will Help Fight Climate Change And Deforestation.

[B3] UNFCCC (1998). The Kyoto Protocol. http://unfccc.int/cop3.

[B4] German Federal Government (1990). Verordnung über die Beschränkung des ordentlichen Holzeinschlags in den Forstwirtschaftsjahren 1990 und 1991. HolzEinschlBeschrV 1990/91.

[B5] IPCC (2005). Decision 16/CMP.1. FCCC/KP/CMP/2005/8/Add3.

[B6] European Commission (2002). The European Framework for Integrated Environmental and Economic Accounting for Forests – IEEAF. Office for Official Publications of the European Communities, Luxembourg.

[B7] Bormannn K, Dieter M, Englert H, Küppers J-G, Rosin A (2006). Die Waldgesamtrechnung als Teil einer integrierten ökologischen und ökonomischen Berichterstattung. Bundesforschungsanstalt für Forst- und Holzwirtschaft, Institut für Ökonomie sowie Statistisches Bundesamt, Umweltökonomische Gesamtrechnung.

[B8] Bundesministerium für Ernährung Landwirtschaft und Forsten (BML) (1992). Bundeswaldinventur Band I-II. Bundesministerium für Ernährung Landwirtschaft und Forsten (Hrsg), Bonn.

[B9] Bundesministerium für Verbraucherschutz, Ernährung und Landwirtschaft (BMVEL) (2004). Die zweite Bundeswaldinventur – BWI^2^. Das wichtigste in Kürze. Bundesministerium für Verbraucherschutz, Ernährung und Landwirtschaft (Hrsg), Bonn.

[B10] Dieter M, Rosin A, Thoroe C (2004). Die forstwirtschaftliche Gesamtrechnung der Bundesrepublik Deutschland im Rahmen des ESVG 1995 für die Jahre 1991 bis 2002. Institut für Ökonomie der Bundesforschungsanstalt für Forst- und Holzwirtschaft, Arbeitsbericht 2004/15, Hamburg.

[B11] Intergovernmental Panel on Climate Change (IPCC) (2003). Good Practice Guidance for Land Use, Land-Use Change and Forestry. http://www.ipcc-nggip.iges.or.jp/public/gpglulucf/gpglulucf.html.

[B12] Umweltbundesamt (UBA) (2005). Deutsches Treibhausgasinventar 1990–2003. Umweltbundesamt Berlin.

[B13] Englert H, Polley H, Sasse V (1996). Schätzung des potentiellen Rohholzaufkommens für den Zeitraum 1996 bis 2020 in Deutschland: Holzeinschlag liegt deutlich unter dem potentiellen Rohholzaufkommen. Holz-Zentralblatt.

[B14] Polley H, Kroiher F (2006). Struktur und regionale Verteilung des Holzvorrates und des potenziellen Rohholzaufkommens in Deutschland im Rahmen der Clusterstudie Forst- und Holzwirtschaft.

[B15] Kollmann F (1982). Technologie des Holzes und der Holzwerkstoffe.

[B16] Dieter M, Elsasser P (2002). Carbon Stocks and Carbon Stock Changes in the Tree Biomass of Germany's Forests. Forstw Cbl.

[B17] Lamlom SH, Savidge RA (2003). A reassessment of carbon content in wood: variation within and between 41 North American species. Biomass and Bioenergy.

